# Supplementary material for: Inflammatory factors and risk of lung adenocarcinoma: a Mendelian randomization study mediated by blood metabolites
Source: Front Endocrinol (Lausanne). 2024 Aug 27;15:1446863. doi: 10.3389/fendo.2024.1446863 (PMC11384989; doi:10.3389/fendo.2024.1446863)
Supplement: Supplementary file 1 [file DataSheet1.zip › supplementary files/S2/GCST90200696/sensitivity-analysis.pdf]

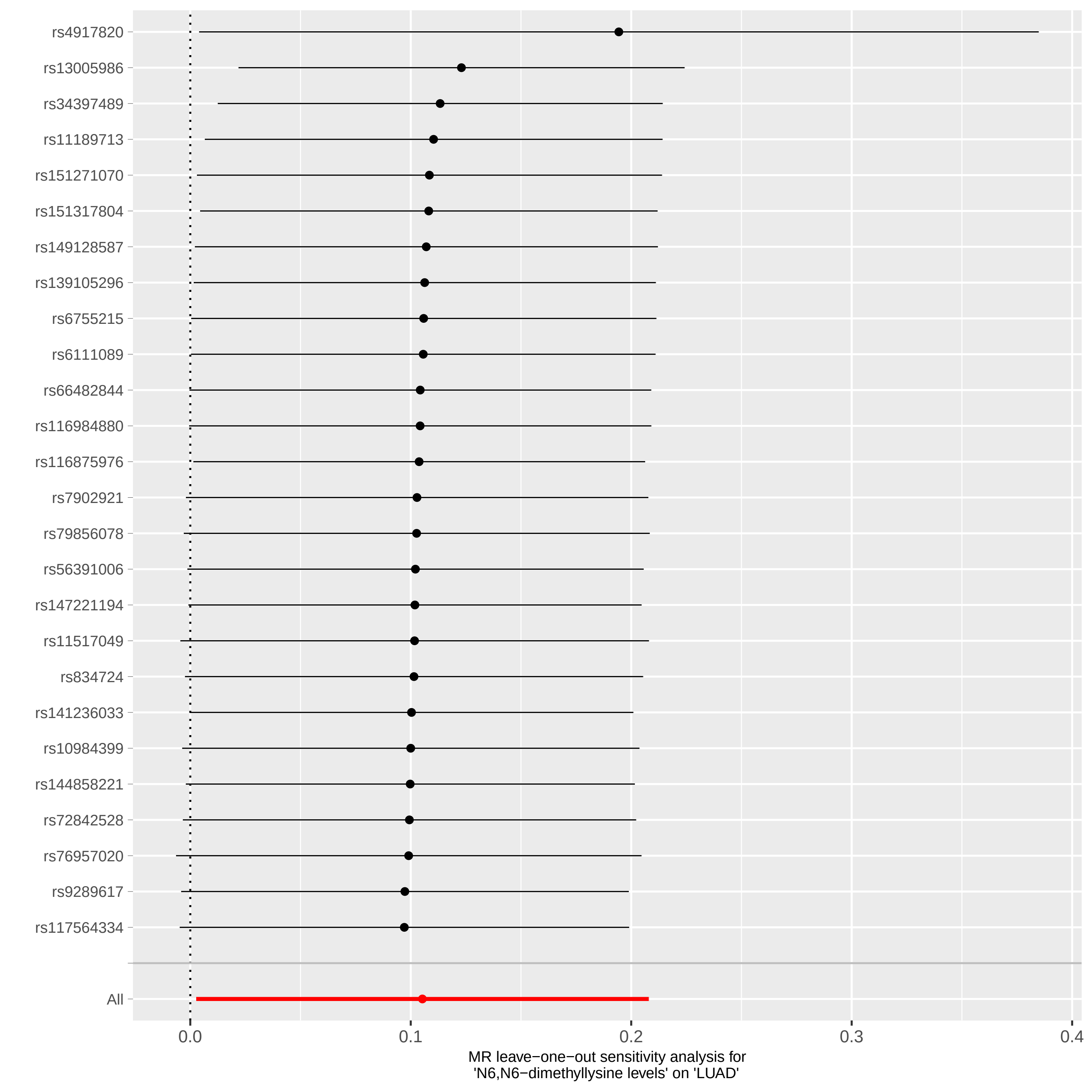

rs4917820
rs13005986
rs34397489
rs11189713
rs151271070
rs151317804
rs149128587
rs139105296
rs6755215
rs6111089
rs66482844
rs116984880
rs116875976
rs7902921
rs79856078
rs56391006
rs147221194
rs11517049
rs834724
rs141236033
rs10984399
rs144858221
rs72842528
rs76957020
rs9289617
rs117564334
All
0.0
0.1
0.2
0.3
0.4
MR leave−one−out sensitivity analysis for
'N6,N6−dimethyllysine levels' on 'LUAD'
